# The Road to Approval: a Perspective on the Role of Icosapent Ethyl in Cardiovascular Risk Reduction

**DOI:** 10.1007/s11892-020-01343-7

**Published:** 2020-10-23

**Authors:** Xiaowen Wang, Subodh Verma, R. Preston Mason, Deepak L. Bhatt

**Affiliations:** 1 Brigham and Women’s Hospital and Harvard Medical School, 75 Francis Street, Boston, MA 02115 USA; 2 University of Toronto, Toronto, Canada; 3 Elucida Research LLC, Beverly, MA USA

**Keywords:** Icosapent ethyl, Eicosapentaenoic acid, Triglyceride, Cholesterol

## Abstract

**Purpose of Review:**

Epidemiological studies have long suggested the cardiovascular benefits of omega-3 fatty acids (OM3FAs). However, until recently, clinical trials using OM3FAs have been largely negative with respect to their cardioprotective effects. In this review, we aim to summarize key clinical trials, examine the clinical benefits of eicosapentaenoic acid (EPA) and potential mechanisms, and review the changes in guidelines and recommendations.

**Recent Findings:**

The Reduction of Cardiovascular Events with Icosapent Ethyl-Intervention Trial (REDUCE-IT) has demonstrated significant cardiovascular mortality benefits of purified EPA ethyl ester, with a 25% relative risk reduction in major cardiovascular events.

**Summary:**

As first of its class to be approved, icosapent ethyl offers a new option to further reduce cardiovascular risks in patients already treated with maximally tolerated statins.

## Introduction

Despite significant advances in our understanding of lifestyle management and pharmacotherapies, cardiovascular (CV) disease remains a major cause of adult mortality both in the US and worldwide [1]. While the wide use of statins in primary and secondary prevention has significantly improved CV outcomes, the quest to identify other therapeutic targets to further reduce CV risk continues [2].

**Fig. 1:**
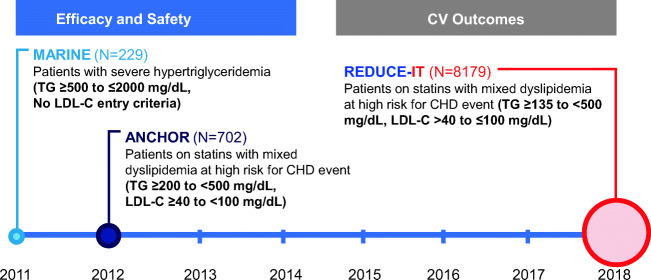
The icosapent ethyl clinical trial program. Eicosapentaenoic acid ethyl ester (icosapent ethyl) was first studied in the MARINE and ANCHOR trials to show that it was effective in reducing triglycerides. REDUCE-IT showed that icosapent ethyl was also effective in improving clinical outcomes and reducing cardiovascular mortality. *CHD* coronary heart disease, *CV* cardiovascular, *LDL-C* low-density lipoprotein cholesterol, *TG* triglycerides

**Fig. 2:**
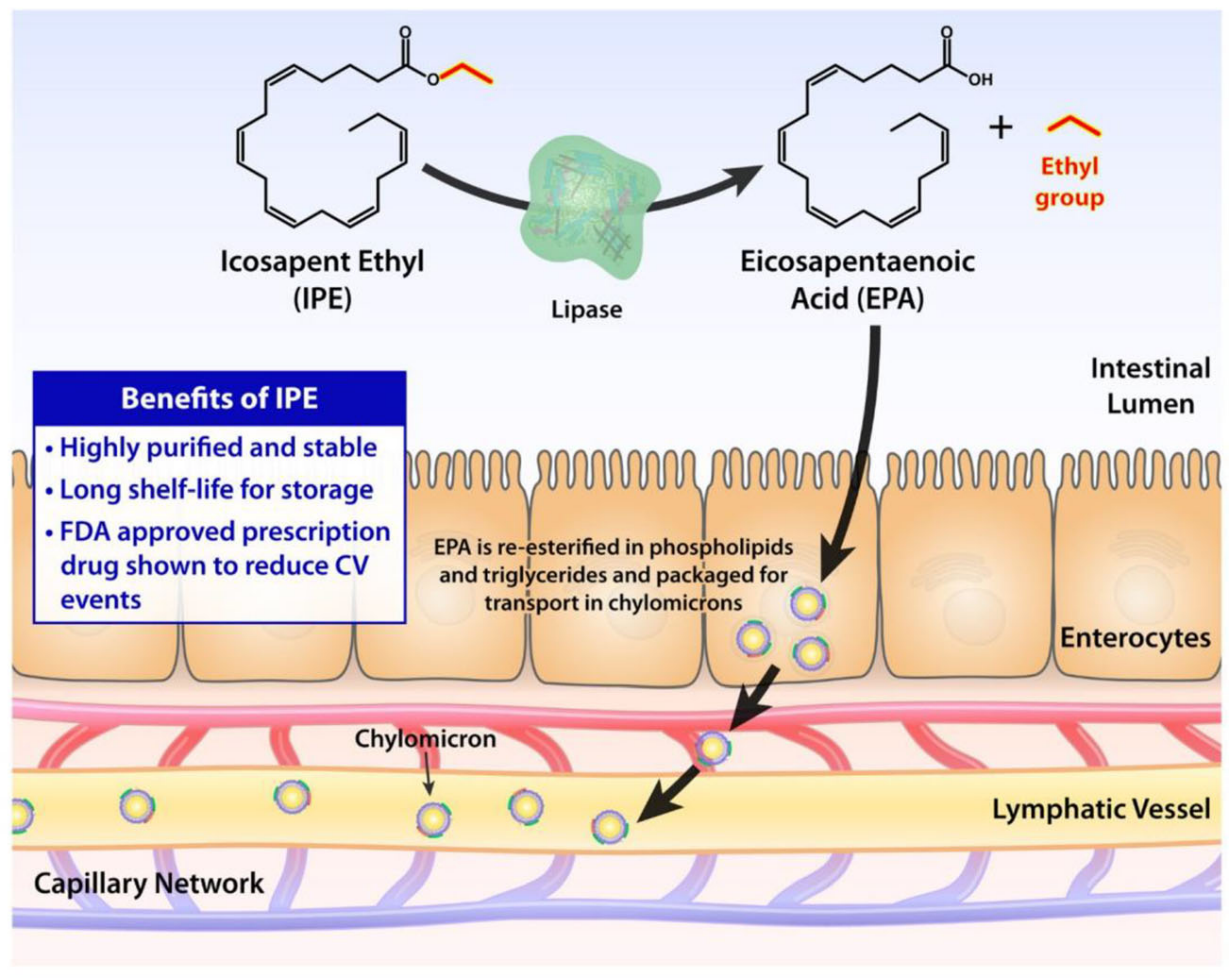
Intestinal processing and absorption of icosapent ethyl (IPE) and its conversion to eicosapentaenoic acid (EPA). *CV* cardiovascular, *FDA* Food and Drug Administration

**Fig. 3:**
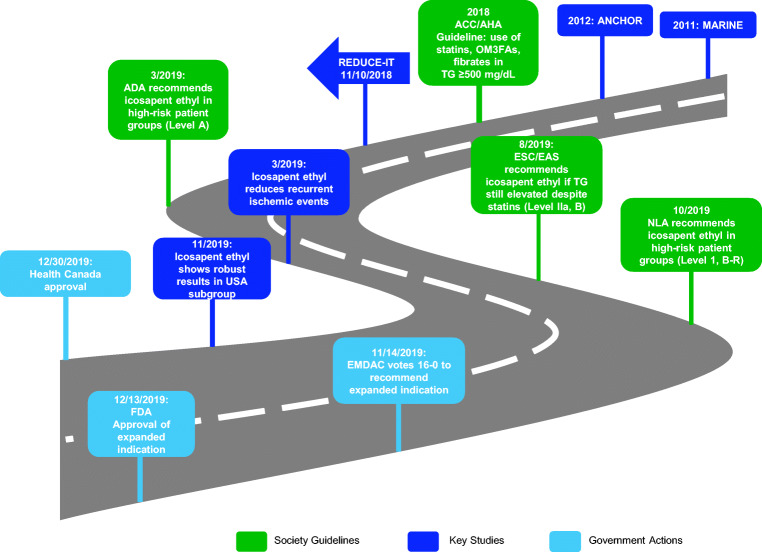
The road to approval: from MARINE and ANCHOR to REDUCE-IT and US FDA and Health Canada approval. Along the way, there have been several key presentations and publications from REDUCE-IT, as well as numerous guideline endorsements. *ACC* American College of Cardiology, *AHA* American Heart Association, *OM3FA* omega-3 fatty acid, *ADA* American Diabetes Association, *EAS* European Atherosclerosis Society, *ESC* European Society of Cardiology, *NLA* National Lipid Association, *EMDAC* Endocrinologic and Metabolic Drugs Advisory Committee, *FDA* Food and Drug Administration

One such therapeutic target is triglycerides. Epidemiological studies suggest that elevated triglyceride levels are associated with increased CV morbidity and mortality, and increased dietary intake of omega-3 fatty acids (OM3FAs) is associated with lower CV mortality [3•, 4–11]. Importantly, this association has been observed at even modest levels of hypertriglyceridemia. Several studies have shown that OM3FAs are effective in lowering triglycerides; however, it had been unclear whether this could translate to any clinical CV benefits [12–14]. In this review, we will summarize key clinical trials that led to the landmark trial REDUCE-IT (Fig. 1), which demonstrated the clinical benefits of icosapent ethyl (Fig. 2) and led to its approval for CV risk reduction (Fig. 3).

### Existing Clinical Trials

Based on epidemiological observations, many prior studies have attempted to examine the clinical effects of lowering triglycerides [3•]. Three major classes of medications have been studied to lower triglycerides or increase HDL: fibrates, niacin, and OM3FAs. While earlier studies showed promising results in using fibrates to improve clinical outcomes before routine moderate and high intensity statin use [15–17], more contemporary trials using fibrates in addition to statins did not demonstrate such clinical benefits [18]. Similarly, studies such as AIM-HIGH and HPS2-THRIVE did not show any clinical benefits of niacin when added to a background of statins [19, 20, 21•].

Many recent studies in OM3FAs showed no significant benefits. The Vitamin D and Omega-3 Trial (VITAL) used a two-by-two factorial design to evaluate the effects of vitamin D3 and OM3FAs at a dose of 1 g per day (containing 380 mg of docosahexaenoic acid [DHA] and 460 mg of eicosapentaenoic acid [EPA]) or placebo in primary prevention of CV disease and cancer [22•]. After randomization of 25,871 participants and a median follow-up of 5.3 years, there was no significant reduction in the rate of major CV events (hazard ratio [HR] 0.92, 95% confidence interval [CI] 0.80–1.06) [22•]. The ASCEND (A Study of CV Events in Diabetes) Study Group evaluated whether OM3FAs improve CV outcomes in patients with diabetes without known CV disease [23•]. The investigators randomized 15,480 participants to receive 1 g of OM3FAs (a mixture of DHA and EPA) or placebo (olive oil) daily. During a mean follow-up of 7.4 years, there was no significant difference in the rate of first serious vascular events (8.9% of patients in the experimental group versus 9.2% in the placebo group, rate ratio 0.97, 95% CI 0.87–1.08) [23•]. Previously, in patients with acute coronary syndrome, OM3FAs in addition to contemporary guideline-directed therapy (including statins and antiplatelets) did not show a decrease in sudden cardiac death or major CV events in the Alpha Omega and OMEGA trials [24, 25].

Two older studies showed clinical benefits of OM3FAs in CV outcomes. In the open-label GISSI-P study, investigators enrolled 11,324 participants with recent myocardial infarction (MI) and randomized them to vitamin E or OM3FAs using a two-way factorial design [26•]. After 42 months of follow-up, the investigators did not find any clinical effects with vitamin E, but found a lower risk of death, nonfatal MI, and stroke in the group that received n-3 polyunsaturated fatty acids (PUFAs). Of the 5666 participants who received n-3 PUFAs (including those who received vitamin E and n-3 PUFAs), 715 met the primary endpoint of death, nonfatal MI, or nonfatal stroke (relative risk [RR] 0.90, 95% CI 0.82–0.99 for two-way analysis); in the n-3 PUFAs only group (2836 patients), 356 patients met the primary endpoint (RR 0.85, 95% CI 0.74–0.98 for four-way analysis) [26•]. Notably, in the GISSI-P study, only 4.7% of participants were on cholesterol-lowering drugs at baseline, which increased to 45.5% at 42 months [26•]. The Japanese study JELIS was also an open-label study [27•]. However, unlike the GISSI-P study in which a combination of EPA and DHA were used, the JELIS study used a highly purified prescription EPA ethyl ester in addition to statins. In JELIS, 18,645 participants with total cholesterol of 6.5 mmol/L (251 mg/dL) or greater were randomized to receive either 1.8 g of ethyl icosapentate (an EPA ethyl ester) with statin or statin only. The study population included both patients for primary and secondary prevention. After a mean follow-up of 4.6 years, the primary endpoint of major coronary events (sudden cardiac death, fatal and nonfatal MI, unstable angina, angioplasty, stenting, or coronary artery bypass graft [CABG]) occurred in 2.8% patients in the ethyl icosapentate group versus 3.5% in the control group (19% relative risk reduction [RRR], *p* = 0.011). The effect was consistent in both the secondary prevention subgroup (8.7% in the ethyl icosapentate group versus 10.7% in the control group, 19% RRR, *p* = 0.048) and in the primary prevention group (1.4% in the EPA group versus 1.7% in the control group, 18% RRR, *p* = 0.13) [27•].

These earlier studies provided important insights for the design of REDUCE-IT (Reduction of CV Events with Icosapent Ethyl-Intervention Trial). First, the dose of the OM3FAs is important to consider. Many of the earlier trials used lower dose of OM3FAs (1–2 g/day), which may not have been sufficient in a Westernized population in which dietary intake of OM3FAs tends to be low. Second, the composition, purity, and stability of OM3FAs merit consideration. The composition of OM3FAs is important, as EPA and DHA have been shown to have different effects on cell membranes [28]. In MARINE and ANCHOR, icosapent ethyl was shown to be effective in reducing triglycerides (without raising LDL-C); however, these trials were not powered for clinical CV outcomes [12, 14].

In REDUCE-IT, 8179 participants were randomly assigned to receive 4 g (2 g twice daily with meals) of icosapent ethyl or matching placebo [29••]. Participants were eligible if they were 45 years of age or older with established CV disease (secondary prevention cohort) or 50 years of age or older with diabetes and at least one additional risk factor (primary prevention cohort). Patients had a low-density lipoprotein (LDL) cholesterol level of 41 to 100 mg/dL and a fasting triglyceride level of 150–499 mg/dL. The protocol initially allowed for a 10% lower triglyceride level (135 mg/dL), due to the inherent variability in triglyceride levels. However, the lower limit was raised to 200 mg/dL during the trial to ensure there would be enough participants in this range of triglycerides. The primary endpoint of CV death, nonfatal MI, nonfatal stroke, coronary revascularization, or unstable angina occurred in 17.2% of participants in the icosapent ethyl group versus 22.0% in the placebo group, a RRR of 25% (HR 0.75, 95% CI 0.68–0.83, *p* = 0.00000001) [29••, 30]. In a subgroup analysis, a similar degree of benefit was seen in patients with diabetes, either with or without baseline CV disease [31].

In a subsequent analysis, icosapent ethyl was shown to not only reduce the first ischemic event, but to also reduce the burden of subsequent and total ischemic events by 30% (RR 0.70, 95% CI 0.62–0.78, *p* < 0.0001) [30, 32••, 33, 34]. A subsequent analysis showed that participants in the icosapent ethyl group had a significantly lower rate of coronary revascularization—both percutaneous coronary intervention (PCI) and CABG [35]. Studies have shown that REDUCE-IT has wide generalizability. Examining an international registry that does not include the US population (which has a high prevalence of hypertriglyceridemia), CLARIFY (ProspeCtive observational LongitudinAl RegIstry oF patients with stable coronary arterY disease) found that 15.5% of patients who had complete data would have been eligible to enroll in REDUCE-IT [36•]. In a Canadian cohort of 196,717 individuals with atherosclerotic CV disease (ASCVD), 25% were eligible for icosapent ethyl based on their lipid profile (well-controlled LDL with elevated triglycerides in the 135–499 mg/dL range) [37]. Furthermore, among the 3146 participants in REDUCE-IT who were randomized in the US, there was a robust reduction in CV risks, with the primary composite endpoint of CV death, nonfatal MI, nonfatal stroke, coronary revascularization, or hospitalization from unstable angina occurring in 18.2% in the icosapent ethyl group versus 24.7% in the control group (HR 0.69, 95% CI 0.59–0.80, *p* = 0.000001) [38••].

Another recent trial, Outcome Study to Assess Statin Residual Risk Reduction with EpaNova in HiGh CV Risk PatienTs with Hypertriglyceridemia (STRENGTH), also examined the effect of OM3FAs in reducing CV morbidity and mortality [39]. In this randomized, double-blind, placebo-controlled trial, 13,086 participants were randomly assigned to Epanova (550 mg of EPA and 200 mg of DHA per 1 g capsule) 4 g once daily or matching placebo, with the primary outcome of time to first event of CV death, MI, stroke, coronary revascularization, or unstable angina [39]. However, at the recommendation of the independent data monitoring committee, the study was discontinued early due to its low likelihood of demonstrating a benefit [40]. This is likely related to the different biological properties of EPA and DHA, as discussed below.

### Mechanisms

**Fig. 4:**
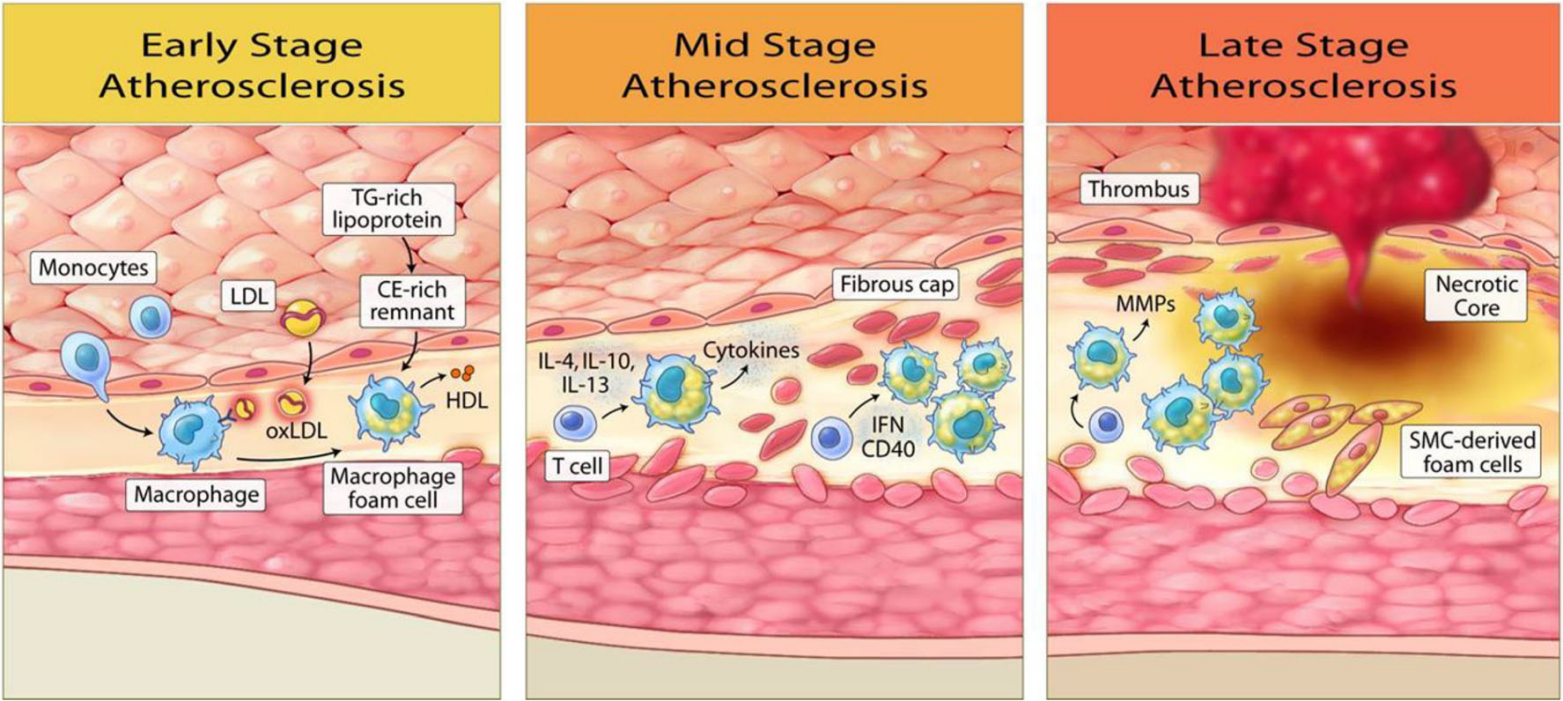
Icosapent ethyl (IPE) modulates the entire atherosclerosis continuum via eicosapentaenoic acid (EPA) and its downstream anti-inflammatory, antithrombotic, and plaque stabilization effects

While REDUCE-IT identified a promising therapeutic target (high-normal to moderately elevated triglycerides) to further reduce CV risk, the exact mechanisms remain unclear but appear to be mediated by EPA and its downstream anti-inflammatory, antithrombotic, and plaque stabilization effects (Fig. 4). The CV benefits from icosapent ethyl are greater than expected from the modest degree of triglyceride lowering in patients with the range of elevated triglycerides in REDUCE-IT, especially in the subgroup of ~ 10% of patients enrolled with normal baseline triglyceride levels. EPA has been shown to have pleiotropic effects that contribute to the lowering of CV risk beyond the effects of triglyceride lowering alone. Clinical studies have shown that treatment with EPA increased fibrous cap thickness, suggesting stabilization of fibroatheroma, and decreased coronary plaque volume [41–44]. In the prespecified interim 9-month analysis of the Effect of Vascepa on Progression of Coronary Atherosclerosis in Persons with Elevated Triglycerides on Statin Therapy (EVAPORATE), icosapent ethyl at 4 g/day was shown to reduce total plaque volume compared with placebo, demonstrating a rather early effect [45]. The final 18-month results of EVAPORATE demonstrate significant reduction in several measures of plaque volume and composition [46••]. EPA has also been shown to reduce macrophage accumulation and local inflammatory markers [41, 42]. Inflammation has been increasingly recognized as an underlying driver of atherosclerosis [47, 48]. EPA is involved in pro-resolving signaling pathways that lead to decreased oxidized LDL (oxLDL) uptake, which plays a crucial role in atherosclerosis [49]. EPA’s observed effects on endothelial function were also a possible contributor to icosapent ethyl’s CV benefits, as indicated by studies demonstrating lower markers of endothelial dysfunction with treatment [50]. In isolated human endothelium, the endothelial benefits of EPA were enhanced when combined with a statin, compared with DHA [28]. In a more recent analysis of REDUCE-IT examining the relationship between EPA level and CV outcomes, patients in the icosapent ethyl group had a higher level of EPA at 1 year and 5 years, and on-treatment EPA level correlated with CV outcomes [51]. Interestingly, the effects of icosapent ethyl were consistent across the full range of baseline triglyceride levels, and the CV benefits were higher than expected for the level of triglyceride reduction, further supporting the notion that the benefits of icosapent ethyl were not simply due to the improvement of other biomarkers.

The differences between EPA and DHA may have contributed to the differences in results between REDUCE-IT and STRENGTH. Studies have shown that EPA and DHA integrate into the cell membrane and interact with cholesterol differently [52, 53••]. DHA has been observed to undergo rapid conformational changes in the cell membrane, promoting cholesterol-rich domains that likely precipitate unstable atherosclerotic plaques, while EPA does not. EPA also appears to have antioxidant effects that are not reproduced with DHA, including under conditions of high glucose [53••, 54]. The antioxidant effects of EPA in both membranes and atherogenic lipoproteins were also superior to other long-chain PUFAs, indicating a favorable combination of carbon chain length and degree of unsaturation for EPA [55]. EPA has been observed to reduce inflammatory markers such as high-sensitivity C-reactive protein, which was not observed in EPA and DHA mixtures [56, 57, 58••]. This anti-inflammatory effect likely has a large contribution to EPA’s CV benefits beyond the level of triglyceride reduction [58••].

Another hypothesized benefit of icosapent ethyl is its antithrombotic effect. OM3FAs, in particular EPA, can integrate into the platelet membrane and replace omega-6 fatty acids [59, 60]. Arachidonic acid (AA), a type of omega-6 fatty acid, plays an integral role in platelet function [59–61]. AA is oxidized by cyclooxygenase (COX)-1 to generate prostaglandins and thromboxanes, including thromboxane A2 that facilitates platelet activation [60, 61]. Instead of generating thromboxane A2, EPA leads to production of thromboxane A3, which has less platelet activation effect than thromboxane A2 [61]. With increased EPA/AA ratio, functional thromboxane A2 decreases, thereby exerting an antithrombotic effect [59, 60]. Indeed, animal models have shown that OM3FAs affect platelet aggregation, thereby reducing thrombus formation [62, 63]. In combination with a statin, EPA also enhances endothelial release of nitric oxide, a potent platelet inhibitor [28]. Several human studies have also demonstrated the antithrombotic effects of OM3FAs. In one study involving 30 participants who were resistant to low-dose aspirin, participants were randomized to receive full-dose aspirin or to receive OM3FAs with low-dose aspirin. Similar to participants treated with high-dose aspirin, those treated with OM3FAs and low-dose aspirin demonstrated reduced platelet activity [59]. In another study, participants with stable coronary disease undergoing PCI were randomized to receive dual antiplatelet therapy (DAPT) with aspirin and clopidogrel, or DAPT plus 1 g of OM3FAs; patients who received OM3FAs had better platelet response to clopidogrel [64]. OM3FAs’ antithrombotic effects may account for the trend towards higher rates of serious bleeding in the icosapent ethyl group (2.7% vs. 2.1%, *p* = 0.06) [29••].

Adverse effects noted in REDUCE-IT included the elevated risks of hospitalization for atrial fibrillation or atrial flutter in patients treated with icosapent ethyl (3.1% in the icosapent ethyl group vs. 2.1% in the control group, *p* = 0.004) [29••]. The underlying mechanism of this observation is unclear. In one animal model, an antiarrhythmic effect was observed with consumption of DHA but not EPA [65]. However, in another animal study, both DHA and EPA were observed to have antiarrhythmic properties, likely due to their effects on sodium currents [66]. Several human studies have attempted to assess the effects of OM3FAs in patients with high risks of arrhythmia, such as patients with implantable cardioverter defibrillators or after cardiac surgery [67–71]. The results of these studies have been mixed, and further research is needed to understand the effects of OM3FAs on atrial and ventricular myocardium [67–71].

### Current Guidelines and Future Directions

The 2018 American College of Cardiology/American Heart Association (ACC/AHA) guidelines on lipids, written prior to the publication of the REDUCE-IT trial results, recognized the elevated ASCVD risks in individuals with hypertriglyceridemia (≥ 175 mg/dL) and recommend the use of statins, OM3FAs, or fibrates in such individuals in addition to lifestyle modification and addressing secondary causes [72]. The 2019 European Society of Cardiology (ESC) guidelines recommend using statins as the drug of first choice in high-risk patients with triglycerides > 200 mg/dL (class I, level B) [73]. These guidelines have already incorporated the findings of REDUCE-IT and recommend the use of icosapent ethyl if triglyceride levels are still elevated despite statin treatment (class IIa, level B) [73]. In the American Diabetes Association (ADA)’s 2020 “Standards of Medical Care in Diabetes,” patients who are already on statins with well-controlled LDL-C but have triglycerides of 135–499 mg/dL, the addition of icosapent ethyl “can be considered to reduce cardiovascular risk” (level A) [74]. The National Lipid Association (NLA) recommends treatment with icosapent ethyl in patients “age ≥ 45 years with clinical ASCVD, or age ≥ 50 years with diabetes requiring medication plus ≥ 1 additional risk factor, with fasting triglyceride 135 to 499 mg/dL on high-intensity or maximally tolerated statin therapy (±ezetimibe)” (class I, level B-R) [75]. Similarly, the Brazilian Society of Cardiology also updated its recommendations to use icosapent ethyl in high-risk patients already on statins with elevated triglycerides (class I, level B) [76]. An AHA Statement on stable coronary artery disease (CAD) in patients with diabetes stated that “Icosapent ethyl is the first non-LDL-focused lipid therapy to demonstrate cardiovascular benefit and should be considered first-line therapy for patients with [type 2 diabetes mellitus] and CAD whose triglycerides remain elevated (> 135 mg/dL) despite maximally tolerated statin and lifestyle changes [77••].”

In addition to its clinical effectiveness, icosapent ethyl was also determined to be cost-effective. The Institute for Clinical and Economic Review (ICER) estimated icosapent ethyl to have an annual wholesale acquisition cost (WAC) of $3699 (rebated cost ~ $1625), lower than ICER’s value-based price benchmark of $6300–$9200 per year [78]. In a separate cost-effectiveness analysis, at a cost of $4.16 per day, icosapent ethyl was shown to be a dominant strategy in the majority of scenario analyses, which is not typically seen with branded medications or devices and previously seen with aspirin or generic statins for secondary prevention [79].

In addition to endorsements from different academic societies and guidelines, icosapent ethyl also received endorsement from government regulatory agencies. Previously, icosapent ethyl was approved as an adjunct to diet to lower triglycerides in adult patients with severe hypertriglyceridemia (triglycerides ≥ 500 mg/dL) to, in essence, reduce the risk of pancreatitis. On November 14, 2019, the Endocrinologic and Metabolic Drugs Advisory Committee (EMDAC) voted unanimously (16-0) to recommend approval of icosapent ethyl for the indication of reducing CV events [80]. On December 13, 2019, icosapent ethyl was approved by the Food and Drug Administration (FDA) to be used as an add-on to maximally tolerated statin therapy in patients with elevated triglycerides (≥ 150 mg/dL, with no specification of the need for fasting) with established CV disease or with diabetes plus two or more CV risk factors [81, 82]. Icosapent ethyl was approved by Health Canada on December 30, 2019. Icosapent ethyl was further endorsed by the US House of Representatives Committee on Appropriations that urged the National Heart, Lung, and Blood Institute (NHLBI) “to support research in this area as well as efforts, particularly through the ‘Know Your Numbers’ campaign, to promote awareness among physicians and patients of the residual cardiovascular risks beyond statin therapy and the importance of taking preventative action to reduce this risk.” [83]

## Conclusion

Despite advances in our understanding of CV risks and use of high-intensity statins, there is still a significant need to further reduce CV risks in patients who are already on optimal statin therapy. Icosapent ethyl provides an efficacious and safe option to reduce triglycerides and further reduce CV mortality and morbidity. While its mechanisms are still to be fully elucidated, the benefits of icosapent ethyl outweigh what would be expected for the degree of triglyceride lowering and are likely attributable to its anti-inflammatory and antithrombotic properties. Icosapent ethyl has been recommended by several professional medical societies. As the first drug of its class to be approved, icosapent ethyl ushers in a new era and provides more options to further reduce residual CV risk in patients with hypertriglyceridemia and other risk factors.
